# A Dominant, Recombination-Defective Allele of *Dmc1* Causing Male-Specific Sterility

**DOI:** 10.1371/journal.pbio.0050105

**Published:** 2007-04-10

**Authors:** Laura A Bannister, Roberto J Pezza, Janet R Donaldson, Dirk G de Rooij, Kerry J Schimenti, R. Daniel Camerini-Otero, John C Schimenti

**Affiliations:** 1 Department of Biomedical Sciences, College of Veterinary Medicine, Cornell University, Ithaca, New York, United States of America; 2 The Jackson Laboratory, Bar Harbor, Maine, United States of America; 3 Genetics and Biochemistry Branch, National Institute of Diabetes and Digestive and Kidney Diseases, National Institutes of Health, Bethesda, Maryland, United States of America; 4 Department of Endocrinology, Utrecht University, Utrecht, The Netherlands; 5 Department of Cell Biology, University Medical Center Utrecht, Utrecht, The Netherlands; Sloan-Kettering, United States of America

## Abstract

DMC1 is a meiosis-specific homolog of bacterial RecA and eukaryotic RAD51 that can catalyze homologous DNA strand invasion and D-loop formation in vitro. DMC1-deficient mice and yeast are sterile due to defective meiotic recombination and chromosome synapsis. The authors identified a male dominant sterile allele of *Dmc1, Dmc1^Mei11^,* encoding a missense mutation in the L2 DNA binding domain that abolishes strand invasion activity. Meiosis in male heterozygotes arrests in pachynema, characterized by incomplete chromosome synapsis and no crossing-over. Young heterozygous females have normal litter sizes despite having a decreased oocyte pool, a high incidence of meiosis I abnormalities, and susceptibility to premature ovarian failure. *Dmc1^Mei11^* exposes a sex difference in recombination in that a significant portion of female oocytes can compensate for DMC1 deficiency to undergo crossing-over and complete gametogenesis. Importantly, these data demonstrate that dominant alleles of meiosis genes can arise and propagate in populations, causing infertility and other reproductive consequences due to meiotic prophase I defects*.*

## Author Summary

About 10%–15% of couples are infertile due to defects in meiosis (the process by which egg or sperm cells containing a single copy of each chromosome are produced). Because studying the genetics of meiosis in humans is difficult, we performed genetic screens in mice and identified a novel mutation in *Dmc1* that causes male-specific infertility due to defects in meiosis. *Dmc1* encodes a key protein required for meiotic recombination; the mutation causes a single amino acid change that prevents genetic exchange, or crossing-over, in males, abolishes its recombination activity, and abrogates the production of sperm. Though heterozygous females are fertile, they have fewer oocytes due to a high incidence of meiosis I abnormalities, and show susceptibility to premature ovarian failure. Importantly, these data demonstrate that dominant alleles of meiosis genes can arise and propagate in populations, and produce meiotic prophase I defects that cause infertility and other reproductive abnormalities.

## Abstract 3

Meiosis occurs in both the male and female germ lines; here the authors describe a mutation that affects only male meiosis, causing sterility.

## Introduction

Genetic recombination occurs in all organisms and is critical for repair of DNA damage, proper chromosome segregation during meiosis, and genetic diversification. Recombination in yeast and mice is initiated by the formation and processing of double-strand breaks (DSBs). Meiotic DSBs are repaired by proteins that mediate homologous strand exchange, mismatch repair, and resolution of recombination intermediates. As these activities are occurring, homologous chromosomes undergo pairing and synapsis, which are completed by the pachytene stage of meiosis. The ability of germ cells to complete meiosis, and to undergo proper segregation of chromosomes in the subsequent meiotic divisions, hinges on the fidelity of these events. Defects in recombination and meiosis have been shown to underlie aneuploidy syndromes such as Downs [1] and azoospermia in men [2].

Our understanding of the genetic control of meiotic recombination in mammals has depended largely on studies of model organisms such as yeast. Mice with null mutations in orthologs of recombination genes often have meiotic defects that are very similar to yeast. However, it is clear that mammals have many genes required for meiosis that do not have orthologs in yeast, and that there are substantial differences between the sexes in the response to mutations in meiotic genes [3–5]. Additionally, mouse null mutations generated by gene targeting of yeast orthologs do not model deleterious hypomorphic or dominant alleles that may occur in human populations.

Because of these issues, and because the genetics of meiosis is difficult to address in humans, we and others have undertaken a forward genetic approach to the identification of novel mutant genes causing infertility in mice [6,7]. An example of the power of this approach was the identification of *Mei1,* a novel vertebrate-specific meiosis gene subsequently implicated in human male infertility [8]. Here, we describe the isolation of an allele of *Dmc1 (Dmc1^Mei11^)* that uncovers remarkable sex-specific properties, unlike a null allele.

*Dmc1* is a meiosis-specific RecA/Rad51 homolog required for recombinational repair of meiotic DSBs. Escherichia coli RecA promotes strand transfer between homologous DNA molecules in an ATP-dependent manner [9], and human DMC1 has intrinsic ATP-dependent strand invasion activity that is stimulated in the presence of the HOP2-MND1 complex [10]. In Saccharomyces cerevesiae and mice of both sexes, DMC1-deficient mutants arrest in late zygonema/early pachynema of meiotic prophase I, with an accumulation of DSBs and defective synaptonemal complex (SC) formation [11–13]. In contrast, *Dmc1^Mei11^* has the unusual property of causing autosomal dominant male sterility. Our studies of this allele provide insight into the biochemical properties of DMC1, underscore stark sexual dimorphism in meiotic recombination and response to errors thereof, and provide experimental proof that autosomal dominant meiotic mutations can arise, propagate, and cause mammalian infertility.

## Results

### *Mei11,* an Autosomal Dominant Mutation, Causes Meiotic Arrest in Male Mice

In previous work, phenotype-driven screens were conducted on pedigrees of mice derived from chemically mutagenized embryonic stem cells [14]. During analysis of a pedigree that was founded with a first-generation (G_1_) daughter of a chimeric male, a fully penetrant, dominant male-specific sterility trait was discovered and mapped to Chr 15 [6] (see Materials and Methods). Based on phenotypic analyses described herein, we labeled this mutation Meiosis defective 11 *(Mei11).* Since *Mei11* can be transmitted only through females, yet the pedigree was initiated by a male chimera, it is possible that the mutation arose not in embryonic stem cells but in the G_1_ female or one of her descendants.

**Figure 1:**
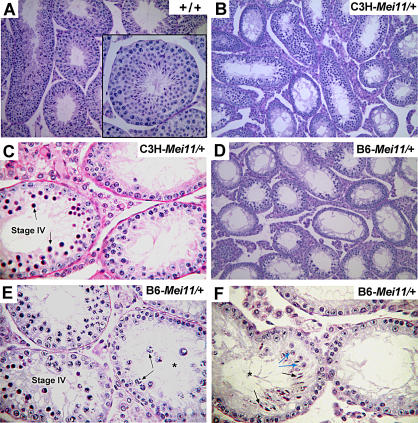
Histopathology of *Mei11* Mutant Testes Paraffin-embedded adult testes sections were stained with hematoxylin and eosin (A–D) or periodic acid–Schiff (E and F). Genotypes are indicated. (A, B, and D) Images are at 20×, except for inset (60×). (C) Dying spermatocytes (arrows) are in an epithelial stage IV seminiferous tubule (60×). (E) Incomplete meiotic arrest. The seminiferous tubule marked with an asterisk contains mid-pachytene spermatocytes (arrows). 60×. (F) A rare tubule (asterisk) containing round spermatids (blue arrows) and elongating spermatids (black arrows). 60×.

We found that spermatogenesis in males heterozygous for *Mei11* in the C3HeB/FeJ (C3H) strain background (≥N3) was arrested in meiotic prophase, with an absence of postmeiotic spermatids (Figure 1A–1C). The most advanced type of germ cells were spermatocytes with nuclear chromatin characteristic of zygotene/pachytene-stage cells (Figure 1B), present in testicular seminiferous tubules at epithelial stage IV, a developmental point at which many meiotic mutants arrest [15]. Failure to progress further led to a massive degeneration of spermatocytes (Figure 1C, labeled tubule). This histological phenotype is indistinguishable from that displayed by *Dmc1* ^−*/*−^ knockout mice [11,12,16]. Interestingly, the meiosis I arrest phenotype was partially modified by genetic background. Heterozygous males in the C57BL/6J (B6) background (N3–N8) were universally sterile, and the vast majority of seminiferous tubules were also arrested at epithelial stage IV (Figure 1D and 1E). However, we observed rare seminiferous tubules containing mid-pachynema spermatocytes (with XY bodies; Figure 1E, arrows) and postmeiotic round and elongating spermatids (Figure 1F, blue and black arrows, respectively).

### *Mei11* Is an Allele of *Dmc1*

We genetically mapped (see Materials and Methods) *Mei11* to a ~2 Mb region containing the meiotic gene *Dmc1* (Figure S1A). Despite the recessive, non–sex-specific nature of null alleles, we sequenced *Dmc1* and identified two consecutive base changes (TG to CC) in all affected *Mei11*/+ males and carrier females (Figure S1B). These correspond to nucleotides 968–969 of the mouse *Dmc1* transcript and cause an alanine to proline change (discussed below). To verify this mutation causes the mutant phenotype, we generated mice containing *Mei11* in *trans* to a *Dmc1* null allele. This resulted in female sterility concomitant with ovarian defects (see below). Furthermore, we found that B6-*Mei11*/*Dmc1^−^* testes underwent complete prophase I meiotic arrest equivalent to that in *Dmc1^−^* ^/*−*^ males (not shown), without signs of postmeiotic differentiation as in B6-*Mei11*/+ males (Figure 1E and 1F). The failure to complement the *Dmc1* null indicates that *Mei11* is a defective allele of *Dmc1* and is likely causative for the infertility phenotype of male heterozygotes. Accordingly, we labeled the *Mei11* mutation *Dmc1^Mei11^.*

### Defective Chromosome Synapsis and DSB Repair in *Dmc1^Mei11^*/+ and *Dmc1^Mei11^/Dmc1^−^* Spermatocytes

**Figure 2:**
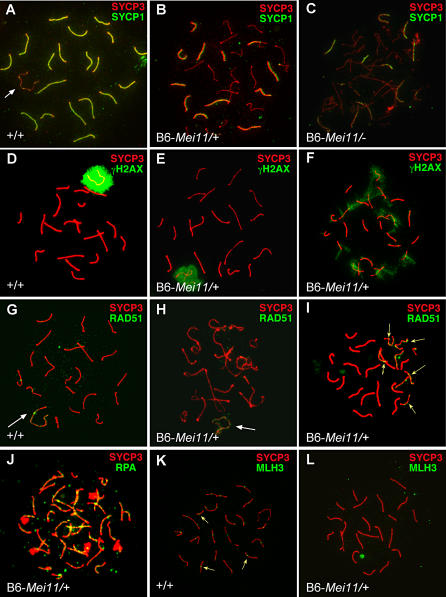
Synaptic Defects, Persistent DSBs, and Lack of Late Recombination Nodules in Mutant Spermatocyte Nuclei Panels show merged images of microspread juvenile (22 to 24 d postpartum) spermatocyte nuclei fluorescently immunolabeled with antibodies as indicated. *Dmc1^Mei11^* is abbreviated as *Mei11;* + or − in genotypes refers to the wt or null alleles of *Dmc1,* respectively. (A) Wt nucleus showing complete synapsis, as judged by overlap of SYCP1/3 staining, resulting in yellow. The white arrow in points to the XY pair. (B and C) Mixture of synapsed and asynapsed chromosomes in aberrant pachytene-like *Mei11*/+ and *Mei11*/*Dmc1^−^* spermatocyte nuclei. (D and E) In wt and pachytene stage B6-*Mei11*/+ nuclei with fully synapsed autosomes, γH2AX decorates the XY body exclusively. (F) γH2AX stains asynapsed chromosomes in a pachytene-like, XY bodyndash;less mutant nucleus. (G and H) RAD51/DMC1 foci are present exclusively on the XY bodies (white arrows) of mid-pachytene nuclei. (I) Persistent RAD51/DMC1 foci are present on cores of presumably unsynapsed chromosomes in an aberrant pachytene-like *Mei11*/+ nucleus (yellow arrows). (J) Abnormal persistence of RPA (yellow) on cores of mutant spermatocytes. (K) MLH3-positive foci, one to two per chromosome, are normally present on cores of mid- to late pachytene wt nuclei (see arrows for examples). (L) MLH3 foci (hence, chiasmata) are absent on meiotic cores of mid-pachytene–like and aberrant pachytene-like (not shown) *Mei11*/+ nuclei.

To characterize the underlying cause of meiotic arrest in mutant spermatocytes, we examined the behavior of meiotic chromosomes throughout prophase I using antibodies directed against diagnostic markers of meiotic chromatin. We monitored chromosome synapsis and SC formation by delineating the distribution of SYCP3, a component of axial/lateral SC elements, and SYCP1, a component of SC transverse filaments that marks synapsed chromosome regions [17,18]. SYCP1 and SYCP3 decorate the axes of all 19 synapsed pachytene stage autosomes in wild-type (wt) spermatocytes, except for the XY bivalent, in which only the pseudoautosomal region (PAR) contains SYCP1 (Figure 2A). While chromosomes in the most advanced *Dmc1^−/−^* nuclei were in a zygonema-like state and devoid of homologous synapsis (unpublished data; [11]), *Dmc1^Mei11^/+* spermatocytes exhibited either a zygotene-like (37% versus 10% in wt controls) or aberrant pachytene-like morphology (62%), containing a mixture of synapsed, partially synapsed, and asynapsed chromosomes (Figure 2B). Consistent with testis histology, we observed rare (1%–2% versus 25% in wt) nuclei with a full complement of synapsed bivalents (Figure 2E). Similar results were obtained with the 125 B6- *Dmc1^Mei11^*/*Dmc1^−^* postleptonema nuclei we examined; 43% were zygotene stage and 57% displayed the aberrant pachytene-like chromosome morphology with regions of SYCP1 staining (Figure 2C) on some chromosome pairs. Thus, these compound heterozygotes progressed further than nulls.

We also monitored synapsis using an antibody against phosphorylated histone H2AX (γH2AX). While serving as a marker of leptotene DSBs [19], H2AX phosphorylation also occurs during pachynema of male meiosis as part of a meiotic signaling pathway involved in transcriptional silencing of unpaired chromatin [20,21]. In wt and (rare) mutant mid-pachytene spermatocytes with fully synapsed chromosomes spermatocytes, γH2AX was present only over the XY body (Figure 2D and 2E). However, in aberrant pachytene-like mutant nuclei, some autosomal meiotic cores stained positively, a pattern indicative of asynapsis (Figure 2F).

To assess the state of recombinant DSB repair in mutant spermatocytes, we characterized the distribution of the RAD51 and DMC1 recombinases by immunolabeling with an anti-RAD51 antibody that recognizes both proteins. These proteins colocalize along meiotic cores in “foci” that are coincident with early meiotic recombination nodules (RNs). We observed no obvious difference in numbers of RAD51/DMC1-positive foci between *Dmc1^Mei11^/+*, *Dmc1^Mei11^*/*Dmc1^−^*, and wt zygotene nuclei (Figure S2). Normally, RAD51/DMC1 foci disappear by mid-pachynema, except along the asynapsed meiotic cores of the XY bivalent (Figure 2G). The rare *Dmc1^Mei11^/+* nuclei that progressed this far had a similar pattern (Figure 2H), but RAD51/DMC1 foci persisted on the asynapsed regions of meiotic cores in aberrant pachytene-like spermatocyte nuclei (Figure 2I, arrows), indicating the presence of unrepaired breaks. Mutant spermatocyte cores also displayed persistent RPA foci, indicating the presence of single-stranded DNA (ssDNA; Figure 2J). When we used a DMC1-specific antibody, we observed approximately half the number of foci in zygotene *Dmc1^Mei11^*/*Dmc1^−^* spermatocytes (average, 52; *N* = 4) compared with wt (average, 113; *N* = 6; Figure S3) and *Dmc1^+/−^* (average, 105; *N* = 5) spermatocytes, suggesting that DMC1^Mei11^ is deficient for RN incorporation (Figure S3).

To determine if crossing-over occurs in *Dmc1^Mei11^/+* spermatocytes, we probed meiotic chromosomes with an antibody against the mismatch repair protein MLH3, a marker of chiasmata [22]. Whereas chromosome cores of wt mid-pachytene spermatocyte chromosomes display one to two MLH3 foci per synapsed homolog (Figure 2K), we saw none on synapsed regions of mid-pachytene–like B6− *Dmc1^Mei11^*/+ chromosomes (Figure 2L).

### *Dmc1^Mei11^*/+ Females Are Fertile but Have a Reduced Oocyte Pool Associated with Chromosome Synapsis Defects

**Figure 3:**
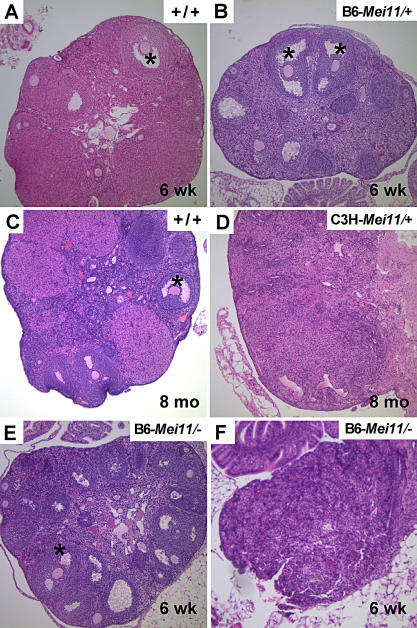
Histopathology of Mutant Ovaries Shown are hematoxylin and eosin–stained paraffin sections of ovaries. The genotypes and ages of the females from which the ovaries were taken are indicated in each panel. Examples of antral-stage follicles with clear oocyte nuclei are indicated with asterisks. *Dmc1^Mei11^* is abbreviated as *Mei11,* and + or − in genotypes refers to the wt or null alleles of *Dmc1,* respectively. The ovary in (F) is more severely affected than from a female of the same genotype in (E).

To examine effects of *Dmc1^Mei11^* on female gametogenesis, we conducted breeding studies, histological analyses of ovaries, and immunocytological examination of meiotic chromosomes. Litter sizes of <6-mo-old *Dmc1^Mei11^*/+ females were normal, regardless of genetic background (Table S1), and the morphologies of young (6 wk) mutant ovaries appeared normal (Figure 3A and 3B). However, a few (4 of 26) C3H congenic females in extended matings (>4 mo) exhibited a premature decline in fertility. The ovaries of these older females were nearly devoid of developing follicles (Figure 3C and 3D). This observation prompted us to quantify the oocyte pool in young, sexually mature mice (6 wk old). As summarized in Figure S4, we noted a depletion of primordial and primary follicle pools in 6-wk-old B6− *Dmc1^Mei11^*/+ females compared with wt animals (by 32% and 31%, respectively). We observed approximately wt levels of more developed follicles, reflecting the ability of ovaries to recruit and mature a “normal” number of oocytes from a smaller resting pool.

*Dmc1^Mei11^*/*Dmc1* ^−^ females were sterile, regardless of genetic background. Interestingly, ovaries of such females fell into two phenotypic classes. Approximately 60% (8 of 13) exhibited a relatively normal ovarian morphology with developing and antral-stage follicles (Figure 3E), albeit fewer than in wt or *Dmc1^Mei11^*/+ (Figure S4). The remaining 40% of paired ovaries (5 of 13 females) were residual and devoid of follicles (Figure 3F), like *Dmc1^−/−^* [11].

**Figure 4:**
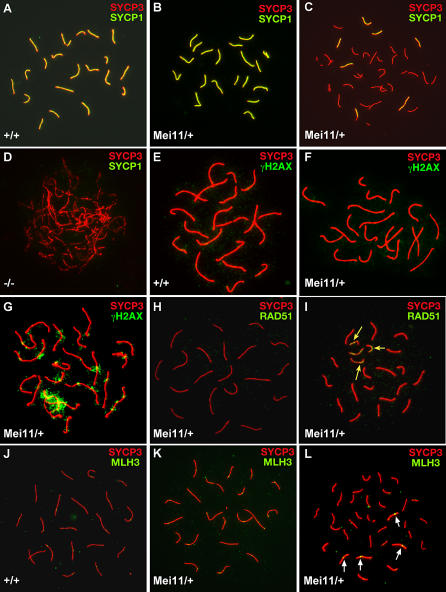
Synapsis and Recombination Defects in Mutant Oocyte Nuclei Microspread oocyte nuclei of embryonic ovaries (17.5 d postcoitus) were fluorescently immunolabeled with the indicated antibodies. All animals were in the B6 background. *Dmc1^Mei11^* is abbreviated as *Mei11,* and + or − in genotypes refers to the wt or null alleles of *Dmc1,* respectively. (A and B) Mid-pachytene nuclei with full synapsis and 20 bivalents. (C) Only a few autosomes are synapsed (yellow staining along chromosomal axes). (D) *Dmc1^−/−^* oocytes have a zygotene-like appearance with very little homolog synapsis. (E and F) Mid-pachytene wt and mutant nuclei with fully synapsed autosomes and no γH2AX staining along the cores. (G) γH2AX present on the cores of an aberrant pachytene-like mutant oocyte. (H) RAD51/DMC1 foci are absent from synapsed pachytene autosomes. (I) Persistent RAD51/DMC1 foci (arrows) are present on (presumably) unsynapsed, but not synapsed, cores of aberrant nuclei. (J and K) MLH3 foci (1–2/chromosome, as yellow spots) are present on wt and some *Dmc1^Mei11^*/+ pachytene cores. (L) In aberrant pachytene-like oocytes, MLH3 foci are present only on synapsed cores (arrows).

To determine whether the reduced oocyte number in mutant adult females was related to recombination and synapsis defects, we examined prophase I chromosomes from embryonic ovaries. Whereas the majority of embryonic day 17.5 oocytes from wt embryos were at mid-pachynema (59% of 63 nuclei examined from 4 embryos, plus 11% in diplonema) with fully synapsed autosomes as indicated by colabeling with anti-SYCP1 and anti-SYCP3 (Figure 4A), only 18% of *Dmc1^Mei11^/+* nuclei (of 131 examined from 3 embryos, plus only 4% in diplonema) had fully synapsed meiotic cores (Figure 4B), whereas the great majority (64%) had either a zygotene morphology or a pachytene-like mixture of completely asynapsed, partly synapsed, and completely synapsed homologs (Figure 4C). *Dmc1^−/−^* chromosomes were mostly asynapsed and zygotene-like (Figure 4D). Interestingly, we observed a unique type (~14% ) of aberrant *Dmc1^Mei11^/+* oocyte nuclei that contained >40 asynapsed pachytene-like meiotic cores (Figure S5). These appear to be oocytes that failed to achieve synapsis and underwent some degree of meiotic core fragmentation as indicated by lack of SYCP1 staining, persistent unrepaired breaks (in the form of RAD51 foci), positive staining of cores for REC8 and STAG3 (not shown), and a lack of centromeric staining (as detected by immunolabeling with CREST antisera) on a subset of mutant oocyte cores. Consistent with the sterility of *Dmc1^Mei11^*/*Dmc1^−^* females, we found no oocytes with a full complement of synapsed bivalents; most were zygotene-like (unpublished data).

We assessed recombination in mutant females cytologically and genetically. Similar to spermatocytes, the fully synapsed chromosomes of normal *Dmc1^Mei11^/*+ mid-pachytene stage oocyte nuclei were devoid of RAD51/DMC1 foci (Figure 4H) and γH2AX staining (Figure 4F), but the unsynapsed cores of aberrant nuclei had persistent RAD51/DMC1 foci (Figure 4I, arrows) and γH2AX signals (Figure 4G), indicative of unrepaired DSBs. The numbers of MLH3 foci along fully synapsed chromosomes of wt and *Dmc1^Mei11^*/+ mid-pachytene oocytes were similar (an average of 25 and 24 foci per wt and mutant nucleus, respectively; Figure 4J and 4K), indicating that crossing-over was normal in this class of mutant oocytes. In aberrant pachytene-like oocyte nuclei, MLH3 foci were present on some meiotic cores but not others, presumably corresponding to synapsed versus unsynapsed bivalents (Figure 4L; arrows). To measure recombination genetically, we crossed C3H-*Dmc1^Mei11^*/+ N12 females to B6 males to create control and heterozygous F1 females, backcrossed them to B6, and genotyped the N2 progeny with polymorphic microsatellite markers along three chromosomes. The overall recombination frequencies were very similar (47.8% versus 46.4%), although the distribution of crossovers on different chromosomes varied (Table 1).

**Table 1:**
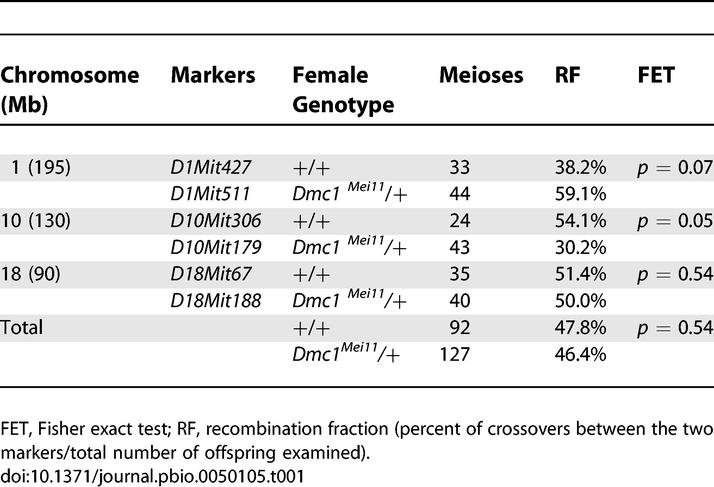
Recombination Rates in Offspring of Wt and *Dmc1^Mei11^/*+ Females

### DMC1^Mei11^ Has Decreased DNA Binding and Is Devoid of Strand Invasion Activity In Vitro

Because of the genetically dominant nature of *Dmc1^Mei11^*, we tested whether DMC1^Mei11^ retains the ability to interact with itself or DMC1 in the yeast two-hybrid system. No qualitative disruption in two-hybrid interactions was observed (Figure S6), suggesting that DMC1^Mei11^ could participate in the assembly of DMC1/DMC1^Mei11^ hybrid polymers.

**Figure 5:**
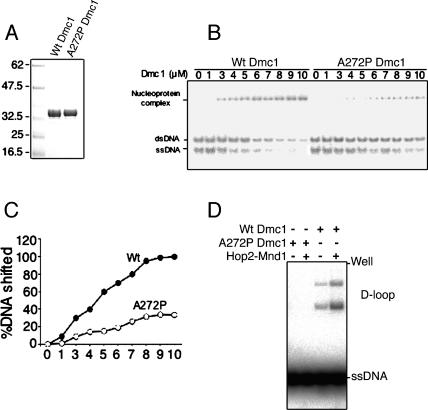
The *Mei11* Mutation Reduces DNA Binding and Abolishes D-Loop Formation All DMC1 protein in this figure is human, and A272P refers to the human protein containing the DMC1^Mei11^ amino acid alteration. (A) Coomassie blue–stained gel showing the bacterially produced and purified wt and mutant proteins. (B) Both wt and mutant show preferential binding to ssDNA. Shown is a gel shift assay in which the indicated amounts of wt or mutant protein (A272P) was incubated with a mixture of single-stranded and double-stranded 100-mer oligonucleotides. (C) The extent of DNA binding for the single-stranded 100-mer. This is a graph of the raw data in (B). (D) The mutant protein is deficient in D-loop formation. The D-loop assay was carried out as described in the absence or presence of cofactor HOP2-MND1 complex.

In vitro, it has been shown that DMC1 nucleates on ssDNA to form extended filaments in an ATP-dependent manner [23–25], and that these nucleoprotein filaments promote a search for DNA homology and strand invasion on an intact duplex (D-loop). Recently it was shown that the HOP2-MND1 complex physically interacts with and stimulates strand invasion mediated by DMC1 [10,26]. The *Dmc1^Mei11^* mutation causes an alanine to proline change at amino acid 272 of DMC1 (A272P), lying within the counterpart of the structurally disordered “Loop 2” (L2) domain of RecA [27] (Figure S1C) that was shown to be involved in DNA binding [28]. To assess the DNA-binding and recombinase activities of DMC1^Mei11^, we constructed an equivalent A272P mutation within the human protein (HuDMC1^Mei11^) and purified it in bacteria (Figure 5A). We analyzed the DNA-binding activity of this mutant by gel shift analysis and measured strand invasion activity using a system optimized for recombinant human DMC1 [10]. Both the wt and mutant protein preferentially bound ssDNA over double-stranded DNA (dsDNA), but the ssDNA-binding activity of the mutant protein was reduced approximately 3-fold (Figure 5B and 5C). Strikingly, mutant HuDMC1^Mei11^ was incapable of catalyzing strand invasion (assayed as D-loop formation), even after addition of the HOP2-MND1 complex (Figure 5D and Figure S7).

To assess possible dominant-negative effects, we measured D-loop activity in experiments where HuDMC1^Mei11^ was mixed with HuDMC1 in stoichemetric quantities. These experiments failed to reveal an inhibition of D-loop activity of the wt protein, either in the absence (unpublished data) or presence (Figure S7) of HOP2-MND1.

## Discussion

### Basis for Sexual Dimorphisms in Meiotic Recombination Uncovered by *Dmc1^Mei11^*

Sexual dimorphism in mammalian gametogenesis exists at several levels, including such basic differences as the timing of meiotic progression and the number of gametes produced, the frequency and distribution of crossovers, and differential checkpoints or checkpoint stringencies [3,29]. In general, sterile mutations in mice that affect chromosome synapsis but not DSB repair confer more severe defects during spermatogenesis than oogenesis. Examples of this are mutations in *Spo11* and *Mei1.* Whereas mutant oocytes can proceed into pachynema and even undergo the first meiotic division, spermatocytes deficient for these genes and DSB repair genes such as *Dmc1* arrest earlier, at the zygotene/pachytene transition [4,30,31]. A more dramatic example of sexual dimorphism is exemplified by animals deficient for the SC component SYCP3. While males arrest at epithelial stage IV (corresponding to mid-pachynema in wt spermatocytes) [5], mutant females are fertile; they produce aneuploid gametes linked to failed chiasmata, causing increased embryonic mortality [32]. These results suggest that meiosis in female mice is more error prone and/or that females are less capable of recognizing and eliminating gametes with meiotic errors, a phenomenon that is relevant to human reproductive biology since aneuplodies that arise in human fetuses have been largely attributed to meiotic errors of maternal origin [33]. The *Dmc1^Mei11^* allele is unique in that oogenesis can be completed in a subset of carrier oocytes despite defects in DSB repair.

The reason for the common arrest point in males is not yet clear. It is possible that different checkpoint signals converge on the same apoptotic pathway, or that apoptosis is caused by a defect shared by the various meiotic mutants. Candidate defects include MSUC (meiotic silencing of unpaired chromatin) that occurs in response to asynapsis, and/or failure of sex chromosome inactivation in mid-pachynema [34], the latter of which may result from excessive autosomal MSUC (P. Burgoyne, personal communication).

The fact that *Dmc1^Mei11^*/+ females were fertile despite exhibiting a high percentage of abnormal meioses, and that most mutant oocytes were capable of normal crossing-over compared with the complete lack thereof in spermatocytes, underscores the stark sexual dimorphism with respect to checkpoint control and recombination mechanisms. A key underlying difference may have to do with the timing of checkpoint activation in meiotic prophase I. For example, an earlier checkpoint activation may occur in spermatocytes before expression of one or more essential components of late RNs, preventing them from being made or modified in a way that is required for late RN formation. Notably, MLH3 foci are apparent prior to pachynema in human oocytes [35], and components of early and late RNs appear earlier in female than male mice in terms of days lapsed from the beginning of meiotic prophase [36]. Later checkpoint activation in oocytes relative to the onset of meiosis might allow more time to resolve recombination/synapsis defects in a subset of mutant oocyte nuclei, allowing them to progress through meiosis and eventually ovulate [37]. Another possibility is that oocytes contain a sufficient or elevated pool of other proteins that can substitute for diminished DMC1 activity. For example, overexpression of *RAD51* or *RAD54* can partially rescue meiosis and sporulation of *dmc1* yeast [38,39].

### Roles of DMC1 in Meiosis and Recombination

Whereas *Dmc1^−/−^* spermatocytes are essentially devoid of homologous synapsis, *Dmc1^Mei11^*/*Dmc1^−^* spermatocytes have a mixture of pachytene-like synapsed homologs and asynapsed chromosomes. Therefore, DCM1^Mei11^ clearly retains some activity that is sufficient to drive some degree of synapsis. Though it is completely unable to catalyze D-loop formation in vitro, the mutant protein might facilitate recombinant activities of other molecules so as to initiate interhomolog pairing. In mammals, DMC1 has been shown to interact with many proteins, including RAD51, RAD54B, BRCA2, MSH4, MND1, and HOP2 [40,41]. Therefore, the DMC1^Mei11^ protein may retain recombinogenic activity in vivo by virtue of protein–protein interactions that are not recapitulated in the in vitro system. Alternatively, other molecules such as RAD51 may be able to drive some amount of interhomolog pairing independently of DMC1, but DMC1^Mei11^ retains an additional, independent role in synapsis per se. Notably, S. cerevesiae is capable of low levels of crossover recombination in the absence of DMC1 [42].

Despite acting in a genetically dominant fashion in vivo, recombinant HuDMC1^Mei11^ did not exert a dominant negative effect on D-loop formation in vitro. One possibility for this discrepancy is that DMC1^Mei11^ forms dominant negative interactions with another key protein(s) in vivo, resulting in defective DSB processing. The mutant protein did not impair the ability of wt DMC1 to catalyze strand invasion in the presence of HOP2-MND1, ruling them out as the target of dominant-negative interactions. However, as stated above, mammalian DMC1 is known to interact with several other meiotic proteins involved in recombination and DNA repair. Since the *Dmc1^Mei11^* mutation decreased DNA binding efficiency in vitro to approximately 30% of wt activity, consistent with a decreased number of DMC1 foci in mutants, it is possible that the dominant-negative interaction may result from binding to a key protein(s) that depends upon DMC1 for localizing to sites of DSB repair.

### Implications for Female Reproduction

In *Dmc1^−/−^* mutant females, oocytes are depleted around birth prior to primordial follicle formation, resulting in residual ovaries that are depleted of ovarian follicles and oocytes [11,12]. The proximal cause of developmental arrest at this stage of oocyte meiosis appears to be the failure to repair DSBs, rather than asynapsis, since genetic elimination of DSBs via mutations in *Mei1* or *Spo11* in DMC1-deficient mice permits further meiotic progression through metaphase I in females [43,44]. Young *Dmc1^Mei11^*/+ females were normally fertile, but had a deficit of primordial and primary stage follicles. Depletion of early-stage follicles in response to unrepaired chromosome breaks and/or asynapsis has also been reported for mice with mutations in *Spo11* and *Sycp3* as well as mouse models of Turner's syndrome [37,43,45]. Overall, these findings indicate that a subset of oocytes was eliminated in mutant females prior to primordial follicle formation, probably leading to the age-related subfertility in a subset of mutant females. Despite the reduction in the oocyte pool, it appears that young *Dmc1^Mei11^*/+ females are able to compensate by recruiting a near-wt number of oocytes for ovulation. This type of compensation has also been reported for mouse models of Turner's syndrome, busulfan-treated rats, and ovariectomized mice [46–48]. The fact that some older females eventually ceased reproduction and displayed oocyte-depleted ovaries appears inconsistent with the notion that the oocyte pool is continually regenerated by circulating stem cells in adult females [49]. Notably, the literature contains a report of a human female with premature ovarian failure that was homozygous for a potential functional mutation in *Dmc1* [50].

Cytological analysis of *Dmc1^Mei11^*/+ oocytes revealed that a significant proportion had unrepaired breaks and asynapsed chromosomes at the time when most oocytes normally reach pachynema and diplonema. It is likely that these meiotic defects (particularly unrepaired DSBs) are sufficient to trigger checkpoint-mediated apoptosis in a subset of oocytes, leading to the observed shortfall of follicles. Some or most of the oocytes that proceeded past this checkpoint were capable of fertilization and development. We do not know if this entire cadre of “successful” oocytes was completely normal, or whether some escaped checkpoint elimination with a subcritical level of aberrations. Future experiments may determine whether mutant oocytes that survive postnatally retain meiotic errors and are subsequently either eliminated prior to fertilization via the spindle checkpoint or alternatively, survive to produce some level of aneuploidy in fertilized oocytes.

Our genetic and functional studies of *Dmc1^Mei11^* provide proof that dominant mutations can arise in a key meiotic recombination gene such as *Dmc1* and cause infertility in mice. Because of the high conservation in the process of meiosis, it is highly likely this could occur in humans with similar consequences (i.e., male infertility, premature ovarian failure, and possible defects in fertilizable oocytes). What makes such a mutation particularly insidious is that it is transmissible through populations via females, who would not manifest any apparent problems until well into their reproductive lifespans.

## Materials and Methods

### Expression and purification of recombinant Dmc1.

Human wt and A272P mutant cDNAs were cloned into the pET-15b vector (Novagen, http://www.emdbiosciences.com) to generate a protein linked to a histidine tag on the amino terminus. The proteins were overexpressed in E. coli BL21 (DE3) and purified using the following series of chromatographic steps: Ni-NTA-agarose (Qiagen, http://www.qiagen.com), heparin (FF Hiprep16/10; Bio-Rad, http://www.bio-rad.com), MonoQ, and Superdex 200. The proteins were concentrated and stored in buffer (20 mM Tris-HCl [pH 7.4], 300 mM NaCl, 10 % glycerol) at −80 °C.

### DNA substrates.

These oligonucleotides were synthesized by MWG Biotech (http://www.mwg-biotech.com): 1, 100-mer, TGTGGAATGCTACAGGCGTTGTAGTTTGTACTGGTGACGAAACTCAGTGTTACGGTACATGGGTTCCTATTGGGCTTGCTATCCCTGAAAATGAGGGTGG; 2, 100-mer, CCACCCTCATTTTCAGGGATAGCAAGCCCAATAGGAACCCATGTACCGTAACACTGAGTTTCGTCACCAGTACAAACTACAACGCCTGTAGCATTCCACA; 3, 60-mer AATGTTGAATACTCATACTCTTCCTTTTTCAATATTATTGAAGCATTTATCAGGGTTATT.

### DNA-binding assay.

Oligonucleotide 1 was 5′ end-labeled with (γ-^32^P)-ATP using standard labeling procedures. The labeled strand was then annealed to the complementary strand (oligonucleotide 2) to create blunt-ended dsDNA and gel purified in 7% polyacrylamide, 1× TAE buffer. Finally, all the duplexes were purified using ion exchange columns (MERmaid Spin Kit; Qbiogene, http://www.qbiogene.com). The single-stranded and the double-stranded 100-mer oligonucleotides were then mixed with either wt or A272P HuDMC1 in reactions containing 20 mM Tris-HCl (pH 7.4), 100 mM NaCl, 10% glycerol, and 1 mM MgCl_2_. The reaction mixtures were incubated at 37 °C for 10 min, and products were analyzed by electrophoresis in 8% polyacrylamide gels in 1× TAE buffer at 5 V/cm for 3 h. The formation of nucleoprotein complexes was quantitated using a BAS 2500 Bio-imaging Analysis System (Fuji Medical System, http://www.fujimed.com).

### D-loop formation.

Supercoiled pUC18 plasmid DNA was purified by CsCl banding. Oligonucleotide 3 (60-mer; 0.82 μM nt) with homology to the pUC18 plasmid was radiolabeled and mixed with supercoiled pUC18 (8 μM bp). Reaction mixtures contained 1 mM DTT, 20 mM Tris-HCl (pH 7.4), 2.5 mM MgCl_2_, and 2 mM ATP with an ATP-regenerating system (7.5 mM creatine phosphate and 30 U/ml creatine kinase). hDMC1 and/or A272P hDMC1 were preincubated with the 60-mer oligo for 5 min, followed by incubation with dsDNA for 15 min at 37 °C. When both HuDMC1 and A272P hDMC1 were incubated together, 100 μg/ml BSA was added to the reaction mixture. Reactions were stopped by the addition of 0.5% SDS and 1 mg/ml proteinase K and incubation for 15 min at 37 °C. Products were resolved on 1% agarose gels in 1× TAE containing 3 mM MgCl_2_. Gels were dried to DE81 paper and analyzed with the BAS 2500 Bio-imaging Analysis System.

The amount of D-loop relative to dsDNA was calculated using this formula: % dsDNA in D-loop = A × 100 × (Ppi_prod,i_ / Ppi_total,i_), where *Ppi_prod,i_* are pixels per inch of oligonucleotide ssDNA in supercoiled dsDNA (plasmid), *Ppi_total,i_* are the sum of pixels per inch of free oligonucleotide ssDNA and oligonucleotide ssDNA in supercoiled dsDNA, and *A* is the molar ratio of oligonucleotide ssDNA with respect to dsDNA in the reaction mixture.

### Mice and genetic mapping.

The stock of mice containing the *Mei11* mutation was derived from a G_1_ daughter of chimera v6.4-C7, as indicated in Table 1 of Munroe et al. [14], who segregated the spermatogonial depletion *(Sgdp)* mutation. *Sgdp,* like *Mei11,* also causes male-specific infertility, but not due to meiotic arrest. At first, only the *Sgdp* phenotype was seen and characterized histologically. Afterwards, in the course of mapping *Sgdp,* males were phenotyped on the basis of testis size, an autosomal dominant pattern of inheritance was evident, and linkage to Chr 15 was obtained [6]. However, upon histological analysis of the affected animals in the mapping cross, we realized that the phenotype was not that of *Sgdp,* but of *Mei11.* We cannot determine whether *Sgdp* and *Mei11* were both present in the original pedigree, or if *Mei11* arose thereafter. Initial genetic mapping indicated that the *Mei11* mutation was segregating with B6 microsatellite alleles on distal Chr 15. For high-resolution genetic mapping, C3H-*Mei11*/+ females were backcrossed to C3H, and 810 offspring were genotyped for the presence of crossovers in the 7.5 Mb *Mei11* critical region, between markers *D15Mit68* and *D15Mit107.* Recombinant males were scored for the mutant phenotype by checking for presence/absence of epididymal sperm or examining testis histopathology. Recombinant daughters were backcrossed to C3H for phenotyping of recombinant sons.

### Genotyping of mice.

The wt allele was identified using the forward primer 5′ TTGTGACCAATCAAATGACAG 3′ and the common reverse primer 5′ CCTAGGATCATCCCCCAAGT 3′. The *Mei11* mutant allele was identified using the forward primer 5′ TTGTGACCAATCAAATGACAC 3′ and the common reverse primer listed above. The *Dmc1^−^* allele was genotyped as described [11].

### Testes cDNA preparation.

Total RNA was isolated from adult mouse testes using RNA midi-prep columns (Qiagen). cDNA was generated from 2 mg of randomly primed RNA using Superscript II (Invitrogen, http://www.invitrogen.com).

### Histological analysis of tissues.

Testes or ovaries were fixed in Bouin's solution, dehydrated, and embedded in paraffin wax. The embedded samples were sectioned at 5 μM and stained with hematoxylin and eosin or periodic acid–Schiff.

### Surface spreading of meiotic chromosomes and immunocytochemistry.

The methodology used for surface spreading and immunolabeling of spermatocyte and oocyte and meiotic chromosomes was as described [7,51]. Primary antibody sources and dilutions used in our experiments are as follows: mouse monoclonal anti-SYCP3 (1:500, ab12452; Abcam, http://www.abcam.com); mouse DMC1 monoclonal (1:250; P. Moens [Department of Biology, York University, Toronto, Ontario, Canada] and M. Tarsounas [University of Oxford, Oxfordshire, United Kingdom]; this antibody was immunodepleted for cross-reactive activity against RAD51 [52]); rabbit anti-SYCP1 (1:1,000, C. Heyting, Wageningen University, Wageningen, The Netherlands); rabbit anti-RAD51 (1:250, PC130; Oncogene Research Products, http://www.emdbiosciences.com/html/CBC/home.html; also cross-reactive with DMC1); rabbit anti-phosphorylated H2AX (γH2AX), serine 139 (1:500, 07–164; Upstate Biotechnology, http://www.upstate.com); rabbit anti-STAG3 (1:1,000; R. Jessberger, Mount Sinai School of Medicine, New York, New York, United States and Dresden University of Technology, Dresden, Germany); rabbit anti-MLH3 (1:400; P. Cohen, Cornell University, Ithaca, New York, United States); rabbit anti-RPA (1:200; C. Ingles, University of Toronto, Toronto, Ontario, Canada), and human anti-CREST (1:500; B. R. Brinkley, Baylor College of Medicine, Houston, Texas, United States). All secondary antibodies conjugated with either Alexa Fluor 488 or Alexa Fluor 594 (Molecular Probes, http://probes.invitrogen.com) were used at a dilution of 1:1,000. All images were taken with a 100× objective lens under immersion oil.

### Ovarian follicle counts.

Paraffin-embedded ovaries (see above) were entirely serial sectioned at 6 μm. Follicle counts were carried out under standard light microscopy, with every tenth section counted. Ovarian follicles were scored and categorized as primordial, primary, secondary, early antral, and antral according to previously described morphological criteria [53]. Only those follicles with clear oocyte nuclei were counted. For a numerical estimate of each follicle class in the entire ovary, counts were then multiplied by a “correction factor” of ten.

## Supporting Information

Figure S1 *Mei11* Is a Mutant Allele of *Dmc1*
(A) Positional cloning of *Mei11.* Schematic representation of mouse Chr 15, showing the relative location of MIT markers used in genetic mapping (small circles) and the number of recombinants/total backcross progeny genotyped.
(B) Sequence traces of *Dmc1* RT-PCR products. Noncoding strand is shown. *Mei11* contains two adjacent nucleotide changes, resulting in double peaks in the heterozygote (marked by bracket; homozygotes cannot be generated due to male sterility).
(C) DMC1 protein structure. A, B, L1, and L2 represent amino acid sequences encoding Walker Motif A, Walker Motif B, and disordered loop regions L1 and L2, respectively. The asterisk indicates the location of the A272 > P mutation in DMC1^Mei11^, which encodes the amino acid immediately proximal to the L2 loop.
(46 KB PDF)

Figure S2 Mutant Zygotene-Stage Spermatocyte Nuclei Contain Normal Levels of RAD51 Foci Wt (A) and mutant (B–C; of indicated genotypes) spermatocyte surface–spread nuclei were colabeled with antibodies against SYCP3 (red) and RAD51 (green). +, wt; −, *Dmc1^−^*; *Mei11, Dmc1^Mei11^*.
(120 KB PDF)

Figure S3 Decreased DMC1 Foci in Mutant Spermatocytes Surface-spread spermatocyte chromosomes were colabeled with antibodies against STAG3 (a component of the meiotic cohesin core that coincides with lateral elements of the SC) and DMC1**.** +, wt; −, *Dmc1^−^*; *Mei11, Dmc1^Mei11^*.
(113 KB PDF)

Figure S4 *Dmc1^Mei11^* Mutant Females Have Reduced Numbers of Primordial and Primary Follicles The graph shows ovarian follicle counts (*y*-axis) from both ovaries of 6-wk-old mutant and wt control littermates as described in Materials and Methods. The types of follicles are indicated on the *x*-axis. *Mei11* = B6− *Dmc1^Mei11^*/+ (*n* = 5 ovaries from three females; shown is the average/ovary); *Mei11*/*−* = B6− *Dmc1^Mei11^/Dmc1^−^* (*n* = 2 ovaries from one female); wt = C57BL/6J (*n* = 6 ovaries from three females). Error bars are standard error of the mean.
(9 KB PDF)

Figure S5 Synaptic Defects, Core Fragmentation, and Persistent DSBs in Mutant Oocytes with >40 Meiotic Cores Genotype abbreviations are as in Figure S4.
(A–B) Mutant oocytes with >40 meiotic cores showing a lack of synapsis.
(C) Wt oocyte nucleus with fully synapsed chromosomes.
(D) Core fragmentation in mutant as suggested by lack of CREST staining (white arrow denotes fragmented core lacking centromeric CREST staining).
(E) Persistent DSBs as identified by persistent RAD51 foci.
(140 KB PDF)

Figure S6 The *Mei11* Mutant Allele Does Not Disrupt DMC1 Homotypic Protein Interactions Shown are yeast two-hybrid interactions of mouse wt and DMC1^Mei11^ proteins, with clones and yeast strains constructed as described in Materials and Methods. All strains were grown on SD-Leu/Trp/His/Ade agar plates. 1–2: DMC1 bait/DMC1^Mei11^ prey; 3–4: DMC1 bait/DMC1prey; 5–6: DMC1^Mei11^ bait/DMC1^Mei11^ prey.
(37 KB PDF)

Figure S7 DMC1^Mei11^ Does Not Affect the Ability of DMC1 to Form D-Loops (A) The ability of human DMC1 (HuDMC1) to form D-loops was analyzed in the presence of varying amounts of HuDMC1^Mei11^ (A272P). HuDMC1 and A272P were incubated together in the indicated ratios (1 mM total protein concentration) with ssDNA, followed by the addition of dsDNA. For mock treatments, an identical volume of A272P-free buffer was added to the reaction (these contained decreasing amounts of wt HuDMC1, identical to that in the WT:A272P lanes).
(B) Graphs of data from experiments of the type shown in (A). These represent an average of four independent experiments.
(38 KB PDF)

Table S1 Fecundity of *Dmc1^Mei11^*/+ Females and Sterility of *Dmc1^Mei11^/Dmc1^−^* Females (19 KB PDF)

### Accession Numbers

The GenBank (http://www.ncbi.nlm.nih.gov/Genbank) accession number for the mouse *Dmc1* transcript is NM_010059.

## Abbreviations

DSBs: double-strand breaks
dsDNA: double-stranded DNA
RN: recombination nodule
SC: synaptonemal complex
ssDNA: single-stranded DNA
wt: wild-type
