# Supplementary material for: A Dominant, Recombination-Defective Allele of Dmc1 Causing Male-Specific Sterility
Source: PLoS Biol. 2007 Apr 10;5(5):e105. doi: 10.1371/journal.pbio.0050105 (PMC1847842; doi:10.1371/journal.pbio.0050105)
Supplement: Supplementary file 1 [file pbio.0050105.sg001.pdf]

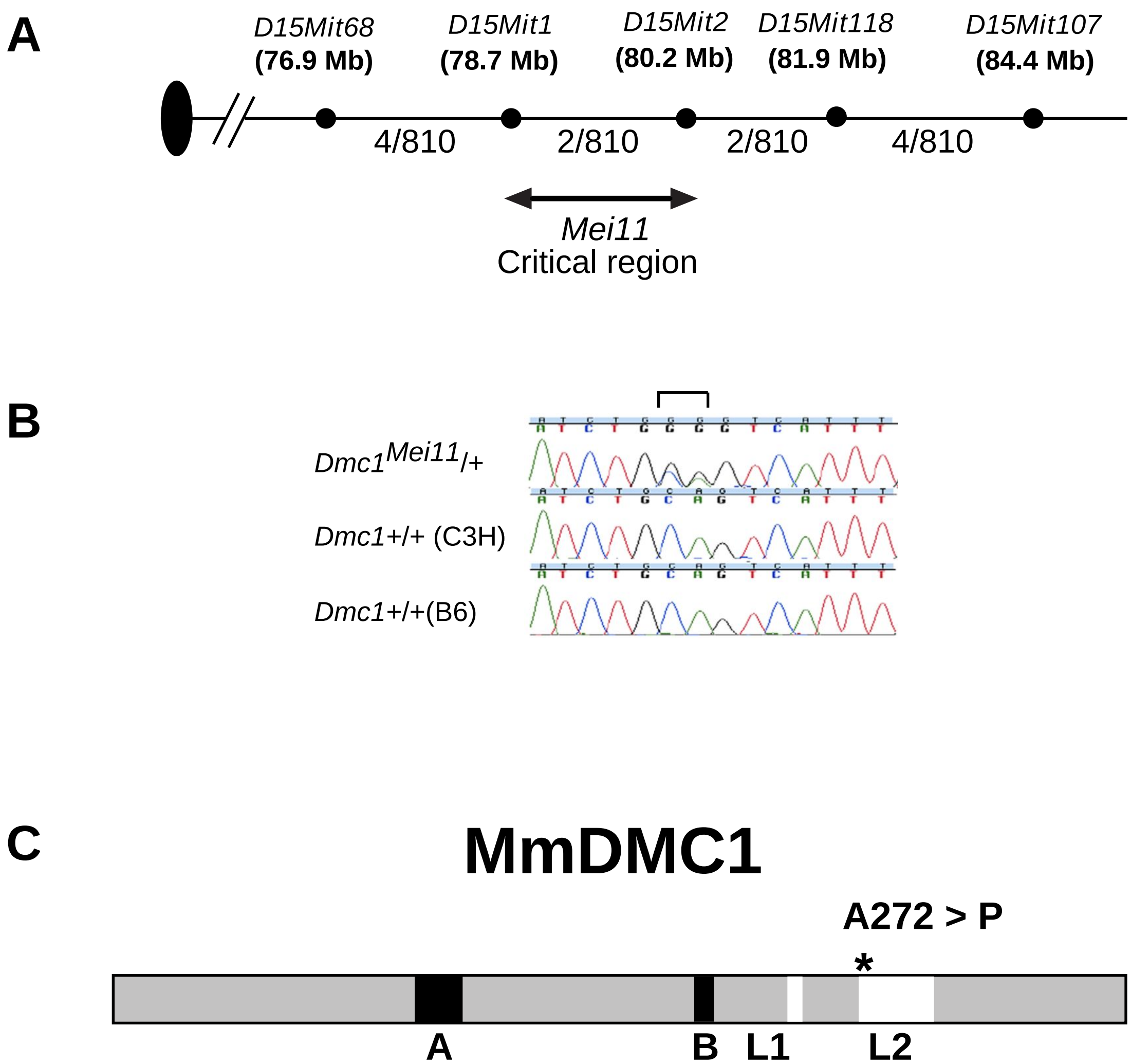
A
D15Mit68
(76.9 Mb)
D15Mit1
(78.7 Mb)
D15Mit2
(80.2 Mb)
D15Mit118
(81.9 Mb)
D15Mit107
(84.4 Mb)
4/810
2/810
2/810
4/810
Mei11
Critical region
B
Dmc1Mei11/+
Dmc1+/+ (C3H)
Dmc1+/+(B6)
C
MmDMC1
A272 > P
*
A
B
L1
L2

Figure S1
