# Supplementary material for: A Dominant, Recombination-Defective Allele of Dmc1 Causing Male-Specific Sterility
Source: PLoS Biol. 2007 Apr 10;5(5):e105. doi: 10.1371/journal.pbio.0050105 (PMC1847842; doi:10.1371/journal.pbio.0050105)
Supplement: Supplementary file 2 [file pbio.0050105.sg002.pdf]

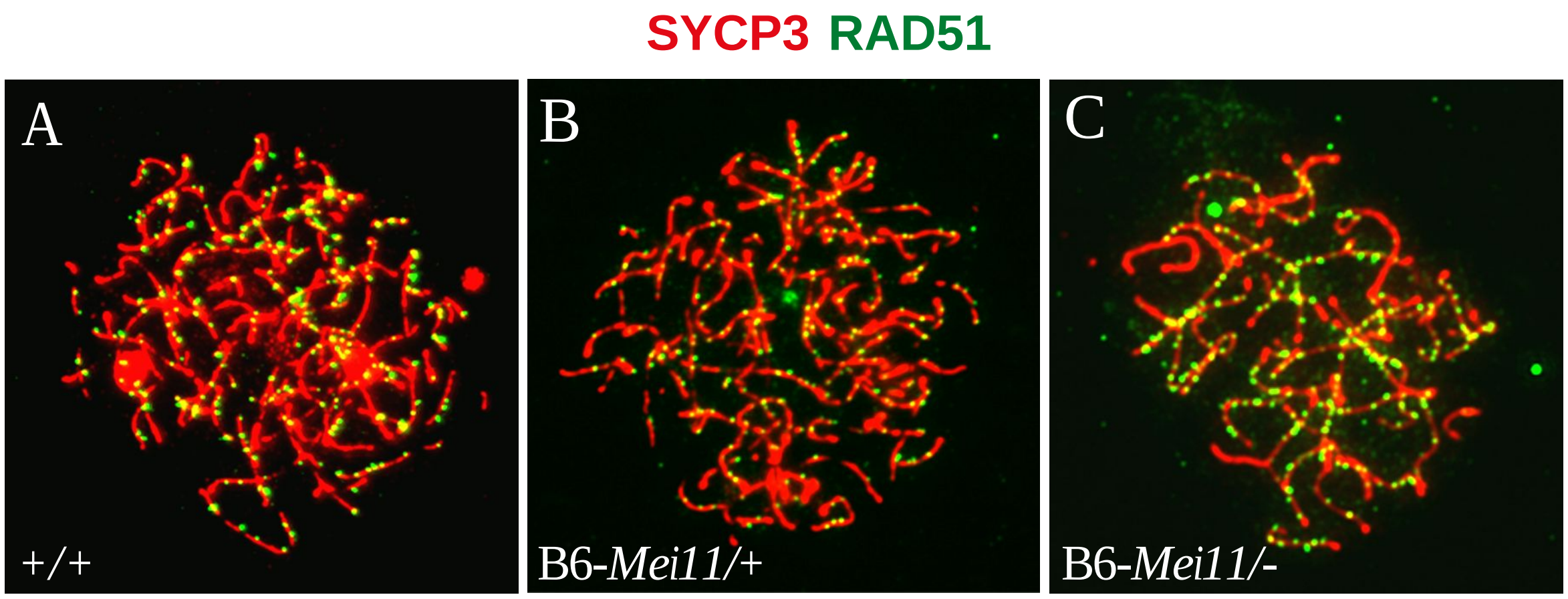

Figure S2
