# Supplementary material for: A Dominant, Recombination-Defective Allele of Dmc1 Causing Male-Specific Sterility
Source: PLoS Biol. 2007 Apr 10;5(5):e105. doi: 10.1371/journal.pbio.0050105 (PMC1847842; doi:10.1371/journal.pbio.0050105)
Supplement: Supplementary file 7 [file pbio.0050105.sg007.pdf]

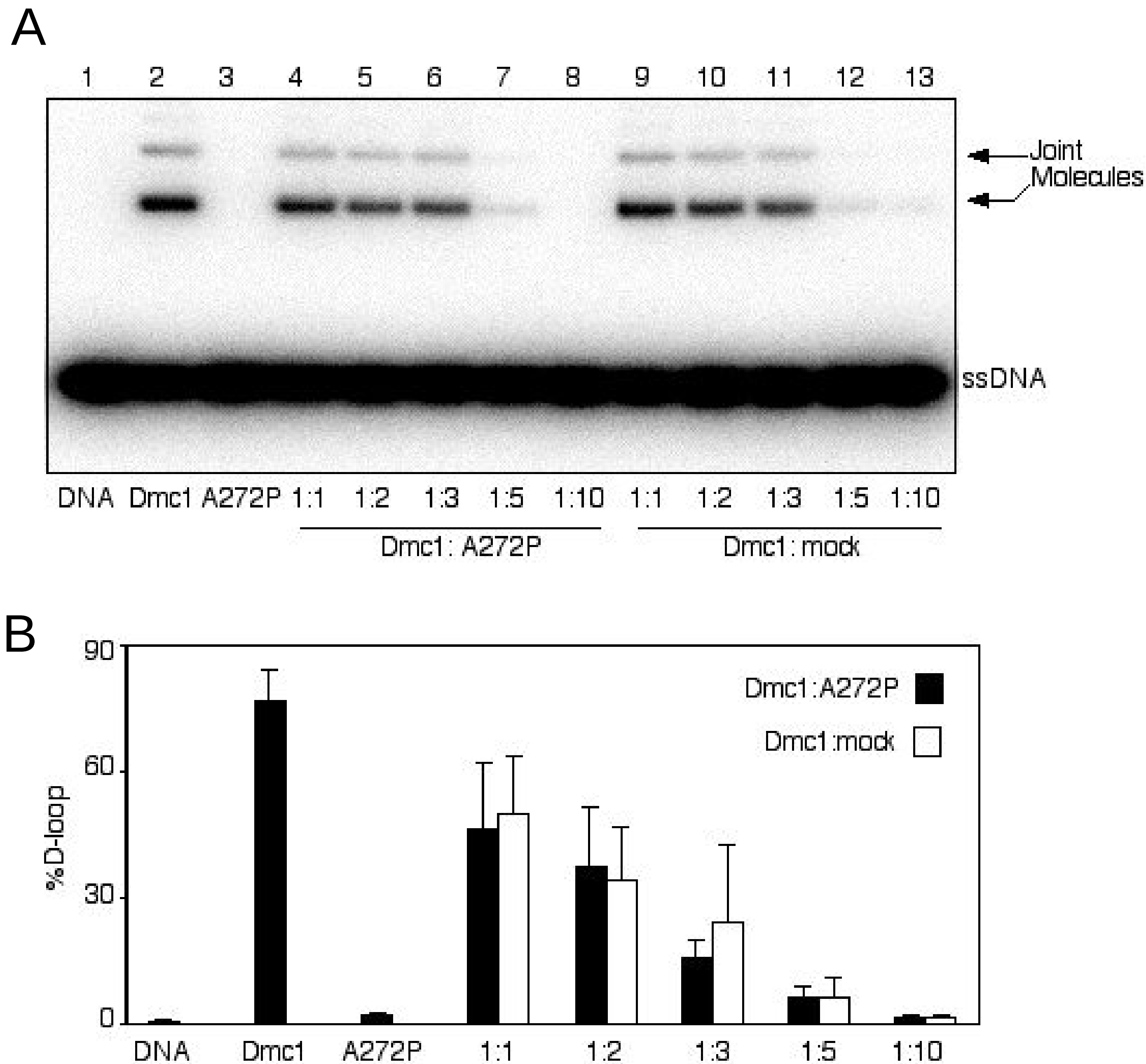
A
1 2 3 4 5 6 7 8 9 10 11 12 13
Joint Molecules
ssDNA
DNA Dmc1 A272P 1:1 1:2 1:3 1:5 1:10 1:1 1:2 1:3 1:5 1:10
Dmc1: A272P
Dmc1: mock
B
90
60
30
0
%D-loop
Dmc1:A272P
Dmc1:mock
DNA Dmc1 A272P 1:1 1:2 1:3 1:5 1:10

Figure S7
