# Supplementary material for: A Dominant, Recombination-Defective Allele of Dmc1 Causing Male-Specific Sterility
Source: PLoS Biol. 2007 Apr 10;5(5):e105. doi: 10.1371/journal.pbio.0050105 (PMC1847842; doi:10.1371/journal.pbio.0050105)
Supplement: Supplementary file 8 [file pbio.0050105.st001.pdf]

Supplemental Table 1. Fecundity of *Dmc1*^{Mei11}/+ females and sterility of *Dmc1*^{Mei11}/*Dmc1*$^{-}$ females

Geno	Strain	# mated	# fertile	Mean size 1st Litter
+/+	B6	3	3	7.0
Dmc1^{Mei11}/+	B6	4	4	7.0
+/+	C3H	4	4	5.3
Dmc1^{Mei11}/+	C3H	14	14	6.5
+/+	(B6xC3H)F1	3	3	8.7
Dmc1^{Mei11}/+	(B6xC3H)F1	5	5	9.0
Dmc1^{Mei11}/*Dmc1*$^{-}$	(B6xC3H)F1	3	0	na
Dmc1^{Mei11}/*Dmc1*$^{-}$	B6	2	0	na
